# Effects of Chest Mobilization and Breathing Exercises on Respiratory Function, Trunk Stability, and Endurance in Chronic Stroke Patients after Coronavirus Disease

**DOI:** 10.3390/medicina59122180

**Published:** 2023-12-15

**Authors:** Yangjin Lee, Yoorim Kim, Donghoon Kim

**Affiliations:** 1 Department of Physical Therapy, Gyeongbuk College, 77 Daehang-ro, Yeongju-si 36133, Republic of Korea; ptyangjin2@naver.com; 2 Department of Physical Therapy, Gimcheon University, 214 Daehak-ro, Gimcheon 39528, Republic of Korea

**Keywords:** COVID-19, diaphragm, chest mobilization, respiratory function

## Abstract

*Background and objectives:* This study investigates the effects of chest mobilization and breathing exercises on respiratory function, trunk stability, and endurance in chronic stroke patients who have contracted coronavirus disease (COVID-19). *Materials and Methods:* Thirty inpatients of a tertiary hospital in South Korea, who had a history of COVID-19 and were diagnosed with stroke within the last 6 months, were randomly assigned to either chest mobilization exercise with breathing exercise (CMEBE) or conservative physical therapy with breathing exercise (CPTBE) groups. The respiratory function, trunk stability, and endurance were measured at baseline and 6 weeks after the interventions. *Results:* Both CMEBE and CPTBE groups showed improvements in respiratory function, trunk stability, and endurance after the intervention (*p* < 0.05). However, the CMEBE group showed significantly greater improvements in forced expiratory volume in 1 s (*p* < 0.05), trunk stability (*p* < 0.05), and endurance (*p* < 0.05) than the CPTBE group. No significant intergroup difference was observed in forced vital capacity and peak expiratory flow. *Conclusions:* The combination of chest mobilization and breathing exercises improved respiratory muscle mobility and endurance, stabilized the trunk, and enhanced balance and the transfer of weight. The findings suggest that this intervention could be beneficial in improving respiratory function and endurance in stroke patients.

## 1. Introduction

Infection with severe acute respiratory syndrome coronavirus-2, which began to spread across the globe around December 2019, mainly causes respiratory illness [1]. Coronavirus disease (COVID-19) presents with mild or overt symptoms in the early stages of infection. It mainly impairs lung function and weakens the body’s ability to cope with other illnesses, thereby exacerbating respiratory and cardiovascular diseases [2]. It can also spread via airborne transmission, particularly in settings such as hospitals, where aerosols are frequently generated, raising an urgent need for pharmaceutical and respiratory interventions to address respiratory concerns [3].

Stroke is a neurological deficit in the central nervous system caused by acute vascular diseases, including intracerebral hemorrhage and cerebral infarction, and is a leading cause of death and physical and mental disabilities worldwide [4]. According to the Korean Statistical Information Service (KOSIS) data provided by Statistics Korea, stroke is one of the top three causes of death in Korea. Research has also shown that the mortality rate due to cardiovascular diseases increases with age, particularly over the age of 65 years [5]. The sequelae of cerebrovascular diseases vary depending on the degree and location of damage on the day of onset, and mostly result in motor, sensory, cognitive, and language impairments and emotional problems [6]. Among these problems, motor function has the greatest impact on daily living, and thus, is the main focus of interventions for stroke patients, to enable them to return to daily life.

Previous studies with stroke patients have mainly focused on developing or investigating the effects of existing interventions that can maximize improvements in motor function while minimizing pain. A recent research trend is focused on developing interventions aimed at improving the physical abilities of stroke patients. For example, one study investigated the effect of task-oriented TheraBand exercises on balance and ambulation in chronic stroke patients [7]; another study investigated the effect of applying the torso pattern of proprioceptive neuromuscular facilitation (PNF) on balance and gait in stroke patients [8]. As shown in these examples, studies targeting stroke patients aim to improve physical functions related to daily life, such as balance and gait abilities.

Damage to the motor cortex and pyramidal tract due to intracerebral hemorrhage or cerebral infarction results in hemiparesis caused by functional abnormalities in related muscles, as well as decreased trunk control ability due to the impairment of abdominal and torso muscle functions [9]. In addition, the combined effects of impaired motor control and the simultaneous contraction of respiratory muscles caused by abnormal muscle tone and movement patterns lead to a decrease in coordination and motor performance of respiratory muscles [10]. Post-stroke hemiparesis is characterized by decreased movement efficiency in motor and posture control due to asymmetric and abnormal alignment of the trunk and chest, leading to decreases in respiratory muscle endurance and changes in the respiratory cycle [11]. Respiratory dysfunction limits the daily activities of stroke patients [12]. Therefore, trunk stability and respiratory function are crucial factors for stroke patients [10]. The impairment of respiratory muscle function in stroke patients leads to a decrease in stability and the deterioration of respiratory muscles [13]. Furthermore, the paralyzed ipsilateral hemidiaphragm rises continuously as its upward and downward movements are decreased during breathing [14]. A study on diaphragm strength in patients with ipsilateral hemidiaphragm paralysis reported that chronic respiratory failure was responsible for the paralyzed ipsilateral hemidiaphragm drawing upwards [15]. It has also been reported that weakened respiratory muscles in hemiparetic patients reduce lung capacity and increase residual volumes of air, resulting in a decrease in maximal respiratory pressures compared with those in healthy individuals [16].

Patients with weakened respiratory muscles experience fatigue and respiratory difficulties, which interferes with their daily functioning, and considerable effort is required to overcome them [13]. In particular, weakened respiratory muscles in the thoracic cage can diminish the ability to cough and expectorate, leading to the accumulation of secretions in the airway and causing complications of various respiratory diseases [17]. Furthermore, while the decreased movement efficiency in stroke patients increases the metabolic demand for oxygen, patients may experience decreased oxygen supply during daily activities due to their reduced inspiratory function, and suffer from oxygen deprivation [18]. This lack of oxygen supply due to respiratory dysfunction can also lead to decreased endurance, resulting in earlier onset of fatigue than usual while walking [19]. With growing awareness of the need to improve respiratory function in stroke patients in order to reduce their mortality and complication rates, research has been ongoing to address their impaired respiratory function [20]. However, there is a lack of interest in stroke patients’ respiratory muscle strength, because there is no clear clinical understanding of the respiratory function, symptoms, and complications of stroke patients [21].

While previous studies have mainly focused on improving stroke patients’ ambulation and balance abilities, research on effective interventions for improving their respiratory function has recently been ongoing because of growing awareness of the importance of improving respiratory function in stroke patients. However, no research has yet been conducted to explore the respiratory function training of chronic stroke patients who have contracted COVID-19 and examine their respiratory function with the aim of improving it using chest mobilization and breathing exercises. COVID-19 has been suggested to be more than a lung infection because it affects the vasculature of the lungs and other organs and increases the risk of thrombosis. Furthermore, patients with stroke are vulnerable to secondary events as a result not only of their poor vascular condition, but also of their lack of access to rehabilitation resources [22]. Its effectiveness on respiratory function, trunk stability, and endurance in stroke patients who have impaired respiratory function after contracting COVID-19 is yet to be verified.

Thus, this study aimed to examine the impact of chest mobilization and breathing exercises on respiratory function, trunk stability, and endurance in chronic stroke patients with a history of COVID-19.

## 2. Materials and Methods

### 2.1. Participations

This study was conducted based on a randomized pre–post experimental design. A therapist with over 3 years of experience performed measurements and analyses using a single-blind method, and the sample size was calculated using statistical power analysis software G*power Version 3.1.9.7 [23], applying an effect size of 0.65, a significance level (α) of 0.05, and a power (1-β) of 0.8. As a result, the required sample size was 30.

This study was conducted from June to September 2022. The study was conducted on 30 inpatients at Y Hospital in Gangwon Province, South Korea, who had a history of COVID-19 and were diagnosed with stroke at least 6 months previously.

The inclusion criteria were: (1) among patients hospitalized for stroke, it had been more than 6 months since they experienced COVID-19; (2) those who did not show symptoms of pneumonia; (3) forced vital capacity (FVC) < 80% and not under special treatment; (4) no heart failure, angina, orthopedic disorders, or other similar conditions; (5) Korean version of the Mini-Mental State Examination (MMSE-K) score ≥ 24; (6) ability to perform the exercise program without problems; (7) ambulation duration ≥ 6 min with or without assistive devices; and (8) understanding the purpose of this study and giving consent to participate.

The exclusion criteria were: (1) difficulty maintaining a sitting or standing position; (2) congenital deformities of the rib cage; (3) inability to perform respiratory function tests due to rib fractures or pulmonary, endocrinal, and orthopedic conditions; and (4) a history of surgery in the chest or abdomen.

All participants received sufficient explanation regarding the purpose and procedures of the study, including precautions, and signed an informed consent form in their own handwriting. The study was conducted after obtaining approval from the institutional review board (IRB no. GU-202207-HRa-06-03-P) (clinical trial registration No. KCT0007806).

### 2.2. Procedure

The participants of this study were randomly assigned to a chest mobilization exercise with breathing exercise (CMEBE) group and conservative physical therapy with breathing exercise (CPTBE) group using an open-source randomizer available at: https://www.randomizer.org (accessed on 3 Sepember 2022).

In this study, a single-blind method was applied, and a total of five assistant researchers were selected based on the following criteria: (1) licensed physiotherapist; (2) clinical experience of at least 3 years; and (3) completion of training on chest mobilization exercise, conservative physical therapy, and breathing exercise programs. To ensure inter-rater consistency and measurement accuracy, the five therapists measured the same patients in groups of three during the study period. The respiratory function, trunk stability, and endurance were measured at baseline (pre-test) and 6 weeks after the intervention (post-test).

### 2.3. Intervention

#### 2.3.1. CMEBE Group

Chest mobilization exercises with breathing exercises were conducted in the CMEBE group.

The intervention was administered for six weeks, five times a week, with two 30 min sessions per day, totaling 60 min of exercise per day. During the chest mobilization exercise, the patient was seated upright, and the therapist placed both hands around the lower part of the chest (ribs 8–11) and performed lateral, backward, and upward extension of the lower part of the rib cage while the patient was taking a deep breath through the nose. The subject was then instructed to exhale slowly through pursed lips while maintaining the extended posture as much as possible. After one set consisting of 30 inhalations, the patient rested for one minute before proceeding to the next set; patients performed three sets in total.

**Figure 1:**
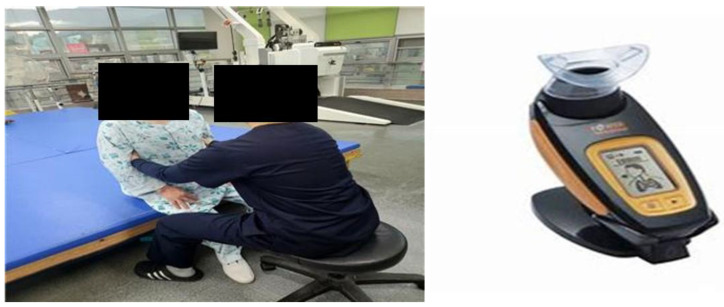
Chest mobilization exercise with breathing exercise.

To integrate breathing exercise into the intervention, a 15 min chest mobilization exercise was followed by a 15 min breathing exercise program, during which the maximum inspiratory pressure (MIP) was measured using K5 (Power breathe, Southam, UK). The patient sat in an upright position with a straight back and waist and exhaled as much air as possible from the lungs through a mouthpiece, followed by a rapid and forceful inhalation, repeating the process 30 times (1 set). After a one-minute rest, the patient proceeded to the next set, performing three sets in total. If the patient felt dizzy or fatigued during the exercise, the exercise was immediately interrupted and resumed after a rest period of comfortable breathing (Figure 1).

#### 2.3.2. CPTBE Group

The CPTBE group performed conservative physical therapy with breathing exercises.

Conservative physical therapy, which includes muscle strengthening, mat training, and stretching, was administered for 30 min, with the difficulty level adjusted according to each patient’s level. The focus was on stability training to increase trunk stability, stretching for body flexibility, and training for muscle strengthening. The therapy was provided by a physiotherapist with over 3 years of experience, and the intervention was administered for six weeks, five days a week, with each session lasting 30 min.

To integrate breathing exercises into the intervention, a 15 min conservative physical therapy was followed by a 15 min breathing exercise program, during which the MIP was measured using K5 (Power breathe, Southam, UK). The patient sat in an upright position with a straight back and waist and exhaled as much air as possible from the lungs through a mouthpiece, followed by a rapid and forceful inhalation, repeating the process 30 times (1 set). After a one-minute rest, the patient proceeded to the next set, and performed three sets in total. If the patient felt dizzy or fatigued during the exercise, it was immediately interrupted and resumed after a rest period of comfortable breathing.

### 2.4. Evaluation

#### 2.4.1. Respiratory Function Assessment

Respiratory function was assessed using an sp70B Spirometer (Contec medical systems, Hebei, China), a portable device for measuring pulmonary functional status in patients with respiratory diseases such as COVID-19 and chronic obstructive pulmonary disease. The device was selected due to its ability to measure FVC and other related parameters using an infrared signal collection mode, as well as its portability, which was necessary for routine tests conducted at the study site. Measurements were captured in a sitting position, and the highest value out of three replicates was selected. The patient sat in an upright position with a straight back and waist and exhaled as much air as possible from the lungs through a mouthpiece, followed by a rapid and forceful inhalation and expiration. Measurements were made in three replicates, and their mean value was calculated. If the patient felt dizzy or fatigued, the measurement process was immediately interrupted and resumed after a rest period of comfortable breathing.

#### 2.4.2. Trunk Stability Assessment

The Trunk Impairment Scale (TIS) is a tool consisting of 17 items that evaluate static and dynamic trunk control and coordination while sitting. The total score ranges from 0 to 23 points, with higher scores indicating better trunk control ability. The inter-rater reliability (r) of the TIS ranges from 0.87 to 0.96, and its intra-rater reliability (r) ranges from 0.85 to 0.99, indicating high reliability and internal validity [24].

#### 2.4.3. Endurance Assessment

Endurance was measured using the 6 min walk test (6MWT) on a 20 m corridor marked at 3 m and 5 m intervals. A ruler was used to measure 1 m intervals. The 6MWT measures the maximum distance a patient can walk in 6 min and is a reliable test for assessing endurance in chronic stroke patients (ICC = 0.94) [25].

### 2.5. Data Analysis

Data analysis was performed using IBM SPSS Statistics 23 Version (SPSS Inc., Chicago, IL, USA). Normality tests were performed on both groups. Homogeneity tests were performed using the chi-squared test for gender and the affected side, and independent t-tests were used for height, weight, age, and MMSE-K scores. Independent t-tests were used to analyze differences between groups, and paired *t*-tests were used to compare pre- and post-intervention scores within each group. Pearson correlation analysis was used to examine the relationship between respiratory function and trunk stability. The statistical significance level was set at α = 0.05 for all data.

## 3. Results

### 3.1. General and Clinical Characteristics of the Patients

Table 1 presents the homogeneity test result for the patients’ general and clinical characteristics.

Table 2 presents the homogeneity test results for respiratory function, trunk stability, and endurance. All three variables showed homogeneity, with no significant differences observed between the groups.

Respiratory function comparison between the CMEBE and CPTBE groups yielded the following results: FVC was 1.88 L in the CMEBE group and 1.62 L in the CPTBE group; the peak expiratory flow (PEF) was 2.56 L in the CMEBE group and 2.33 L in the CPTBE group; the mean trunk stability score was 15.60 in the CMEBE group and 16.02 in the CPTBE group; the 6 min walk distance was 181.02 m in the CMEBE group and 179.00 m in the CPTBE group. There were no significant intergroup differences in respiratory function, trunk stability, and endurance (Table 2).

### 3.2. Changes in Respiratory Function before and after the Intervention

Respiratory function was analyzed using a diagnostic sp70B spirometer (Contec Medical Systems, Hebei, China). Upon completion of the 6-week intervention, pre–post changes in respiratory function were compared between the CMEBE and CPTBE groups.

Both groups showed a significant difference in FVC between the pre- and post-intervention measurements: an increase of 0.76 L from 1.88 L to 2.64 L (*p* = 0.001) in the CMEBE group, and an increase of 0.51 L from 1.62 L to 2.13 L in the CPTBE group. However, on comparing the extent of change between the two groups (0.76 L vs. 0.51 L), there was no statistically significant intergroup difference in FVC (Table 3).

Both groups showed a significant pre–post difference in forced expiratory volume in 1 s (FEV1): an increase of 1.11 L from 2.94 L to 3.24 L (*p* = 0.001) in the CMEBE group and an increase of 0.39 L from 1.72 L to 2.11 L in the CPTBE group (*p* = 0.002). Comparing the extent of change between the two groups (1.11 L vs. 0.39 L), there was a statistically significant intergroup difference in FEV1 (*p* < 0.05) (Table 3).

Both groups showed a significant pre–post difference in PEF: an increase of 1.09 L from 2.56 L to 3.65 L (*p* = 0.002) in the CMEBE group and an increase of 0.91 L from 2.33 L to 3.23 L in the CPTBE group (*p* = 0.002). However, comparing the extent of change between the two groups (1.09 L vs. 0.91 L), there was no statistically significant intergroup difference in PEF (Table 3).

### 3.3. Changes in Trunk Stability before and after the Intervention

Trunk stability was assessed using the TIS. Both groups showed a significant pre–post difference in the mean TIS score: an increase of 3.32 points from 15.60 to 18.92 in the CMEBE group (*p* = 0.001) and an increase of 2.19 points from 16.02 to 18.21 in the CPTBE group. When comparing the extent of changes between the two groups, the CMEBE group showed a significantly greater improvement in trunk control ability than the CPTBE group (*p* < 0.05) (Table 4).

### 3.4. Changes in Endurance before and after the Intervention

Endurance was measured using the 6MWT. After 6 weeks of intervention, the changes in endurance before and after the intervention were compared between the CMEBE and CPTBE groups. Both groups showed a significant pre–post difference in the 6 min walk distance: an improvement of 20.98 m from 181.02 m to 202.00 m in the CMEBE group (*p* = 0.001) and an improvement of 3.32 m from 179.00 m to 182.32 m in the CPTBE group (*p* = 0.001). Comparing the extent of changes between the two groups, the CMEBE group showed a significantly greater improvement in endurance than the CPTBE group (*p* < 0.05) (Table 5).

## 4. Discussion

This study was conducted to investigate the effects of an intervention combining chest mobilization and breathing exercises on respiratory function, trunk control, and endurance in stroke patients who contracted COVID-19. Thirty stroke patients who had recovered from COVID-19 were randomized into the CMEBE and CPTBE groups. Respiratory function, trunk control, and endurance were measured in both groups before and after the intervention to evaluate the pre–post changes within each group and between the two groups.

Stroke results in significant changes in respiratory function due to impaired trunk control [4], as well as changes in the respiratory mechanism or impaired pulmonary function [26]. Reduced lung capacity is an important contributing factor for restrictive pulmonary dysfunction in stroke patients [2]. Such restricted physical capacity and resulting physical inactivity induce a decrease in respiratory muscle efficiency and an increase in rib cage asymmetry, leading to restricted movement. Treatment of this condition comes with a high dependence on steroid therapy, which is known to have serious side effects, such as pneumonitis [26]. Therefore, we conducted a comparison study with CMEBE (chest mobilization + breathing) and CPTBE (physiotherapy + breathing) groups, in which respiratory function, trunk control, and endurance were measured and within-group changes and between-group differences were examined. In this section, the effects of the interventions on respiratory function, trunk control, and endurance in stroke patients are discussed.

In this study, the respiratory function of stroke patients was evaluated by measuring FVC, FEV1, and PEF. FVC measures the maximum volume of air that a patient can forcefully exhale after inhaling as deeply and rapidly as possible. FEV1 measures the volume of forcefully exhaled air over one second while measuring FVC. PEF measures the flow of air during a forced expiration after inhaling the maximum possible amount of air. Significant increases in FVC, FEV1, and PEF were observed in both the CMEBE and CPTBE groups, with the CMEBE group showing greater improvements in FEV1 and PEF. Respiratory intervention methods are categorized into voluntarily controlled breathing and breathing using resistance-training devices. A study using a respiratory resistance-training device in chronic stroke patients reported significant improvements in FVC, FEV1, and PEF [27]. The K5 respiratory resistance-training device was used in this study to improve respiratory function in chronic stroke patients, yielding similar results. Both the CMEBE and CPTBE groups achieved significant increases in FVC and FEV1. The FVC and FEV1 values of the CMEBE group were calculated at 70% and 65%, respectively, indicating mild restrictive pulmonary dysfunction.

Restricted physical capacity and resulting physical inactivity can lead to decreased respiratory muscle efficiency and increased rib cage asymmetry, resulting in restricted movement. To address this problem, it is necessary to apply an appropriate respiratory exercise program that directly or indirectly enhances the mobility and endurance of respiratory muscles [19]. Endurance was evaluated using the 6MWT. When comparing the pre–post changes in endurance between the CMEBE and CPTBE groups, the CMEBE group showed a more marked statistically significant difference compared with the CPTBE group. The comparison of pre–post changes between the two groups with a focus on the intervention methods revealed that both groups achieved significant improvements, but the CMEBE group showed a greater improvement than the CPTBE group. This finding is similar to that of a study with 40 chronic stroke patients, in which significant improvements in the 6MWT were confirmed in both the experimental group (proprioceptive neuromuscular facilitation + breathing exercise) and the control group (conservative physiotherapy + breathing exercise) [28]. In a study using respiratory resistance-training with visual feedback, the experimental group showed a significant pre–post improvement in the 6MWT in within-group comparisons and a significant difference in a between-group comparison [29], as shown by the CMEBE group in this study. This suggests that the improvement in respiratory muscle mobility and endurance contributed to improving the walk distance in the 6MWT. The slight significant improvement in walk distance observed in the CPTBE group may have been due to the effects of strength training, mat exercises, and stretching exercises.

The CMEBE group showed a significant increase in FEV1, to a greater extent than the CPTBE group, supporting the results of a previous study [27]. Previous studies have demonstrated positive effects of chest mobilization exercises on chest expansion and trunk stability function in stroke patients [30,31]. Additionally, chest mobilization exercises have been reported to improve respiratory muscle activity and function, resulting in improved respiratory function [32], as well as trunk control ability in stroke patients [30]. The trunk control ability of stroke patients has been reported to be associated with their gait and endurance [24]. In this study, both groups showed a significant increase in trunk stability, which is consistent with previous research [33]. A study on improving endurance in stroke patients using chest mobilization and breathing exercises found that inspiratory muscle training with chest expansion was a particularly effective intervention for improving respiratory muscle strength and endurance without causing damage to the diaphragm and auxiliary respiratory muscles [34]. Another study showed that a six-month intervention using respiratory muscle training improved respiratory function and muscle endurance in stroke patients [35]. In this study, combining chest mobilization and breathing exercises induced patients to perform controlled breathing by themselves, which had the effect of stabilizing the trunk by improving the weakened respiratory muscle mobility and endurance of stroke patients who had contracted COVID-19. The enhanced trunk stability may have contributed to significant improvements in walk distance in the 6MWT by enhancing balance and ability to transfer weight.

The outcome values of this study showed partial increases and significant differences, rather than overall improvements. There are three limitations to this study that should be noted. First, as this study was conducted only in inpatients and outpatients of Y Rehabilitation Hospital in Gangwon Province, the results may not be generalizable to all stroke patients. Second, as this study recruited patients with reduced respiratory function due to COVID-19 among chronic stroke patients, the results may not be generalizable to all chronic stroke patients. Third, this study could not control for factors that may affect the measurement results of stroke patients other than intervention variables, including daily life and psychological factors. Overcoming these limitations in future research and applying the intervention method of combining chest mobilization and breathing exercises proposed in this study to stroke patients could more effectively improve their respiratory function, trunk stability, and endurance. Additionally, it is necessary to conduct follow-up studies to examine the maintenance of the intervention’s effects and further test its effectiveness.

## 5. Conclusions

This study found that the CMEBE was effective in improving respiratory function, trunk stability, and endurance in chronic stroke patients who have contracted coronavirus disease. Our results indicate that CMEBE training can be considered as a potential method to improve the respiratory function, trunk stability, and endurance in chronic stroke patients who have contracted coronavirus disease. Diversified CMEBE will need to be developed for broader application of the combined approach as a therapeutic intervention for the functional recovery of chronic stroke patients who have contracted coronavirus disease. This study has significance in that it proposed methods for intervention in chronic stroke patients who have contracted coronavirus disease who need long-term for treatment by presenting methods for intervention for the improvement.

## Figures and Tables

**Table 1:** General characteristics of patients (*n* = 30).

Parameters	CMEBE Group (*n* = 15)	CPTBE Group (*n* = 15)	t/x^2^	*p*
Gender				
Male	9 (60.0%)	6 (40.0%)	0.032	0.785
Female	6 (40.0%)	9 (60.0%)		
MAS			0.826	0.421
G0	6 (40.0%)	6 (40.0%)		
G1	5 (33.3%)	6 (40.0%)		
G1+	4 (26.7%)	3 (20.0%)		
Affected side			0.248	0.211
Right	6 (40.0%)	5 (33.3%)		
Left	9 (60.0%)	10 (66.7%)		
Diagnosis			0.146	0.862
Infarction	7 (46.7%)	4 (26.7%)		
Hemorrhage	8 (53.3%)	11 (73.3%)		
Age (years)	59.93 ± 12.48	55.21 ± 0.33	0.322	0.578
Height (cm)	165.93 ± 7.48	162.13 ± 5.35	0.321	0.572
Weight (kg)	68.20 ± 6.88	60.93 ± 5.63	0.237	0.213
MMSE-K	27.13 ± 1.60	27.47 ± 1.46	−0.851	0.251

Mean ± standard deviation, CMEBE group: chest mobilization exercise with breathing exercise group, CPTBE group: conservative physical therapy with breathing exercise group.

**Table 2:** Heterogeneity test of respiratory function, trunk stability, and endurance of study participants (*n* = 30).

Parameters	CMEBE Group (*n* = 15)	CPTBE Group (*n* = 15)	t/x^2^	*p*
Breathing function				
FVC (ℓ)	1.88 ± 0.76	1.62 ± 0.51	0.652	0.416
FEV1 (ℓ)	2.94 ± 1.11	1.72 ± 0.39	1.231	0.122
PEF (ℓ)	2.56 ± 1.09	2.33 ± 0.91	0.326	0.562
Trunk stability				
TIS (score)	15.60 ± 3.32	16.02 ± 2.19	−0.982	0.274
Endurance				
6-MWT (m)	181.02 ± 20.89	179.00 ± 3.32	−0.458	0.325

Mean ± standard deviation, CMEBE group: chest mobilization exercise with breathing exercise group, CPTBE group: conservative physical therapy with breathing exercise group, FVC: forced vital capacity, FEV1: forced expiratory volume in one second, PEF: peak expiratory flow, TIS: trunk. Impairment Scale, 6-MWT: 6-Minute Walk Test.

**Table 3:** A comparison of the pre–post respiratory function between (*n* = 30).

Measures	CMEBE Group (*n* = 15)	CPTBE Group (*n* = 15)	t	*p*
FVC (ℓ)				
Pre	1.88 ± 0.76	1.62 ± 0.51		
Post	2.64 ± 0.52	2.13 ± 0.66		
Change	0.76 ± 0.24	0.51 ± 0.15	1.453	0.335
t(p)	−3.172 (0.001 **)	−2.518 (0.001 **)		
FEV1 (ℓ)				
Pre	2.94 ± 1.11	1.72 ± 0.39		
Post	3.24 ± 0.63	2.11 ± 0.32		
Change	1.11 ± 0.52	0.39 ± 0.07	−0.521	0.042 *
t(p)	−3.836 (0.001 **)	−3.447(0.002 **)		
PEF (ℓ)				
Pre	2.56 ± 1.09	2.33 ± 0.91		
Post	3.65 ± 0.64	3.24 ± 0.76		
Change	1.09 ± 0.45	0.91 ± 0.15	1.325	0.219
t(p)	−3.452 (0.002 **)	−8.485 (0.001 **)		

CMEBE group: chest mobilization exercise with breathing exercise group, CPTBE group: conservative physical therapy with breathing exercise group, FVC: forced vital capacity, FEV1: forced expiratory volume in the one second, PEF: peak expiratory flow, * *p* < 0.05, ** *p* < 0.001.

**Table 4:** A comparison of pre–post trunk stability (*n* = 30).

Measures	CMEBE Group (*n* = 15)	CPTBE Group (*n* = 15)	t	*p*
TIS (score)				
Pre	15.60 ± 3.32	16.02 ± 2.19		
Post	18.92 ± 2.31	18.21 ± 2.13		
Change	3.32 ± 1.01	2.19 ± 0.06	−2.414	0.017 *
t(p)	−1.231 (0.013 *)	−2.218 (0.011 *)		

CMEBF group: chest mobilization exercise with breathing exercise group, CPTBE group: conservative physical therapy with breathing exercise group, TIS: trunk. Impairment scale, * *p* < 0.05.

**Table 5:** A comparison of pre–post endurance (*n* = 30).

Measures	CMEBE Group (*n* = 15)	CPTBE Group (*n* = 15)	t	*p*
6-MWT (m)				
Pre	181.02 ± 20.89	179.00 ± 3.32		
Post	202.00 ± 15.23	182.32 ± 2.12		
Change	20.98 ± 5.66	3.32 ± 1.20	0.326	0.042 *
t(p)	−7.536 (0.001 **)	−9.018 (0.001 **)		

Mean ± standard deviation, CMEBE group: chest mobilization exercise with breathing exercise group, CPTBE group: conservative physical therapy with breathing exercise group, 6-MWT = 6-Minute Walk Test, * *p* < 0.05, ** *p* < 0.001.

## Data Availability

Upon reasonable request, the corresponding author is willing to provide the data and materials supporting the results of this study.
